# Supplementary material for: Bradyrhizobium diazoefficiens cultures display phenotypic heterogeneity
Source: ISME Commun. 2024 Mar 28;5(1):ycaf054. doi: 10.1093/ismeco/ycaf054 (PMC11996625; doi:10.1093/ismeco/ycaf054)
Supplement: Supplementary_figures_ycaf054 [file supplementary_figures_ycaf054.pdf]

Supplementary material for

Phenotypic heterogeneity in *Bradyrhizobium diazoefficiens* USDA 110

Sukhvir K. Sarao[1], Armaan K Sandhu[1], Ryan L. Hanson[1], Tanvi Govil[2], and Volker S. Brözel[1,3]

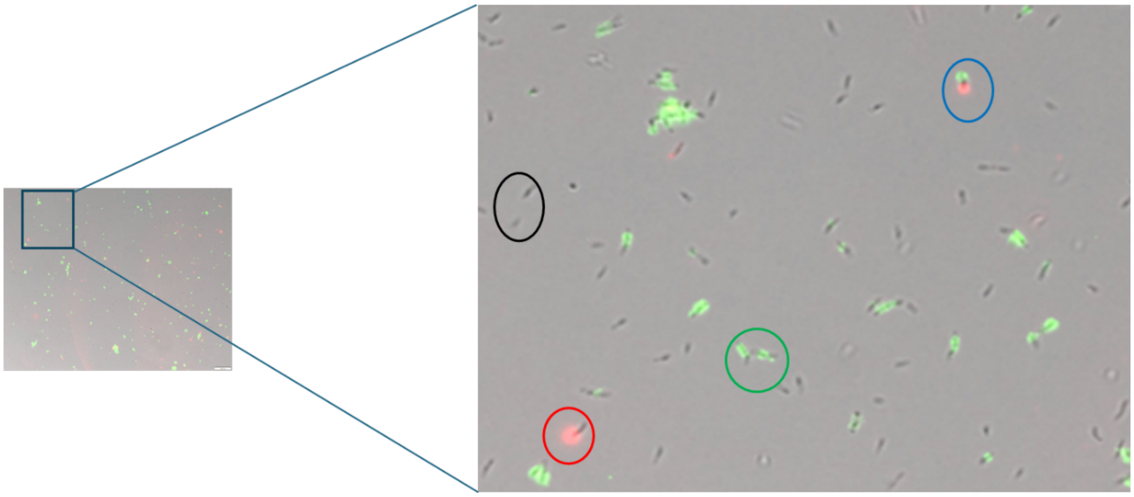

Figure S1. Exponential phase cells of *B. diazoefficiens* USDA 110 stained with Soybean Agglutinin (SBA labelled with fluorescein) and Wheat Germ Agglutinin (WGA labelled with rhodamine), revealing four surface phenotypes.

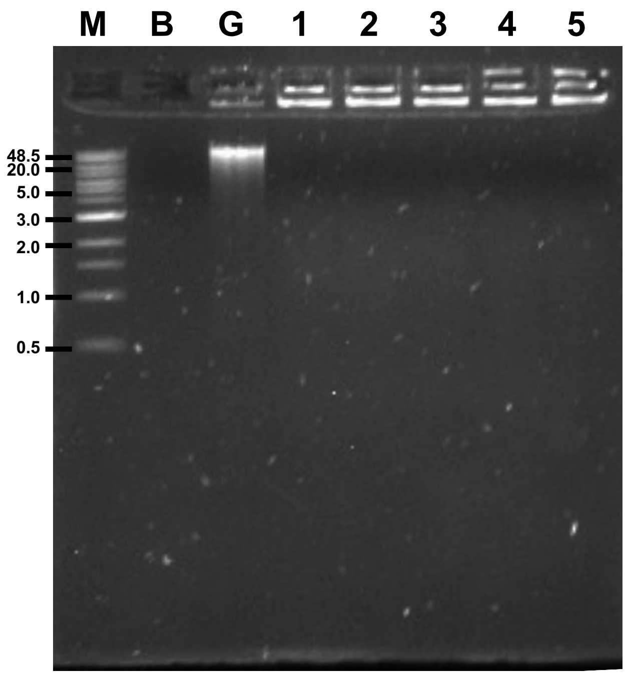

Figure S2. eDNA visualization using agarose gel (left to right): M = molecular mass marker, B = water blank, G = *B. diazoefficiens* genomic DNA, 1 = Early exponential, 2 = Mid Exponential, 3 = Transition, 4 = Early stationary, 5 = Late stationary phase. eDNA was resolved by adding cell pellets to agarose gel wells 1 - 5 as described previously (Vilain et al. 2009).

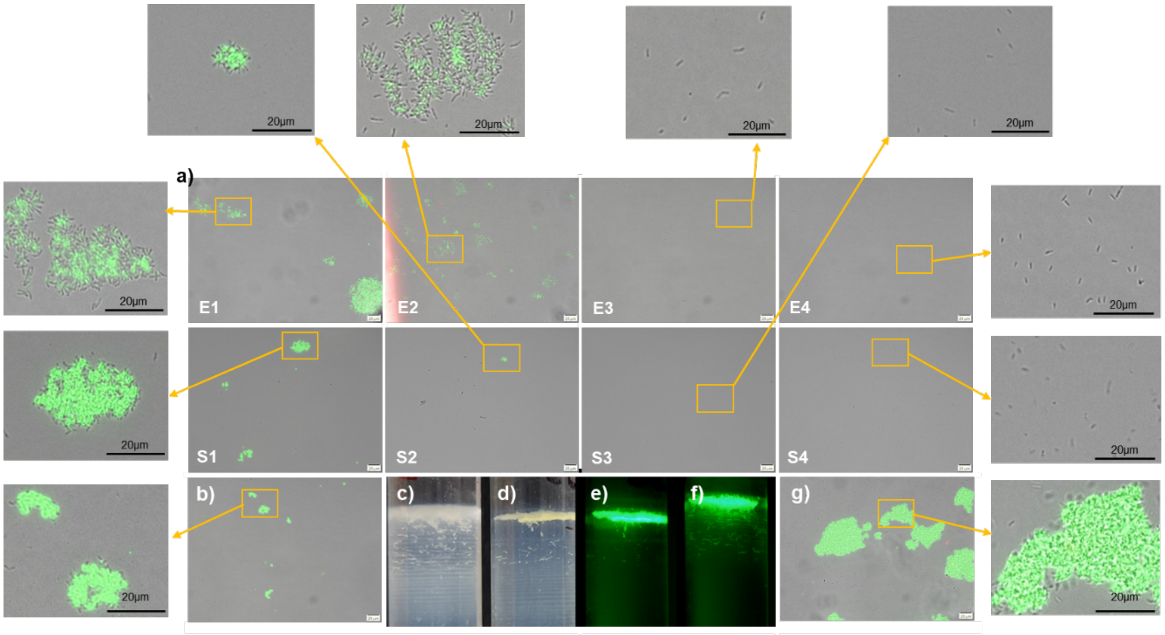

Figure S3. Lectin (Soybean Agglutinin) binding profile of exponential and stationary phase fractions as viewed by fluorescence microscopy (a), and with boxed areas enlarged. Lectin treated unseparated exponential population is shown in panel (b). Gradients of exponential phase populations without (c) and with (d,e) prior treatment with Soybean Agglutinin viewed by illumination and (c,d), and fluorescence (e). Stationary phase populations pretreated with SBA and viewed by fluorescence (f), and fluorescence microscopy of the top fraction showing association of cells by the lectin (g).

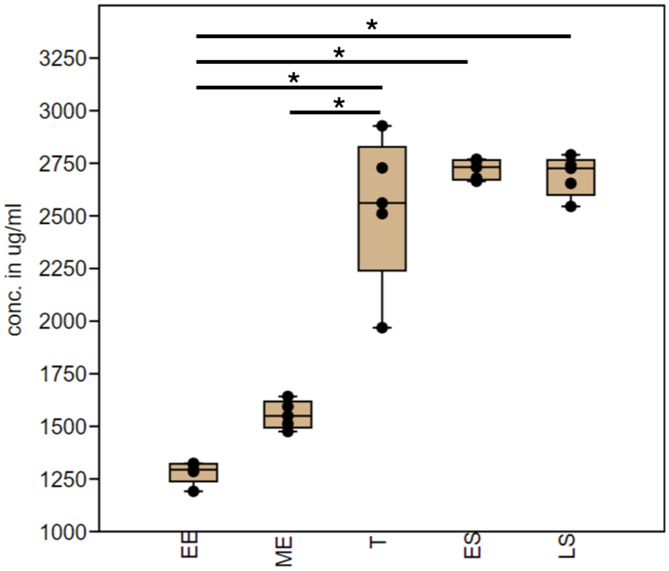

Figure S4. EPS quantified in various phases of liquid *B. diazoefficiens* USDA 110 cultures i.e. early exponential (EE), mid exponential (ME), transient (T), early stationary (ES) and late stationary (LS) (Dunn's post hoc, $p < 0.05$). EPS was extracted and quantified as described previously (Sandhu et al. 2023).

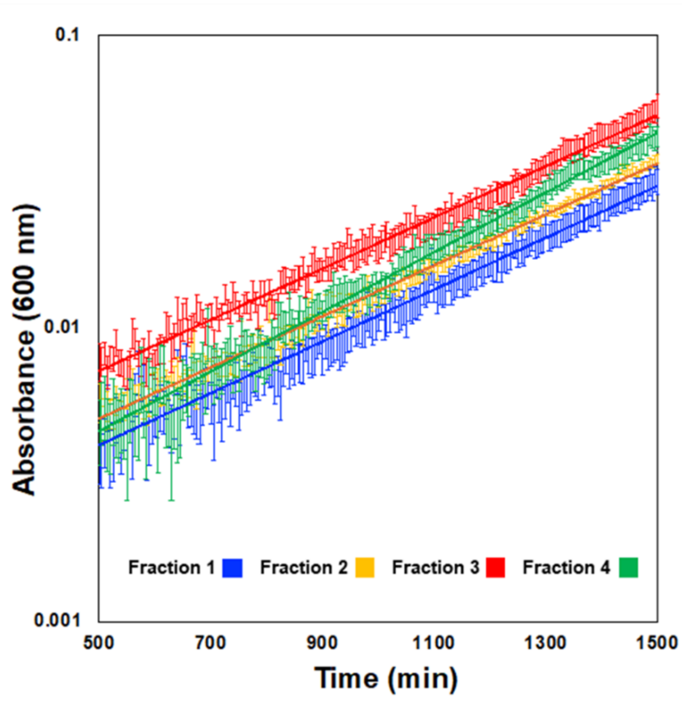

Figure S5. Growth of *B. diazoefficiens* USDA 110 exponential phase fractions. Fractions of exponential phase culture were diluted into fresh PSY-arabinose broth in 96 well plates (n=16) and the absorbance measured every 10 min while shaking at 30 °C. Error bars show one standard deviation of the mean.

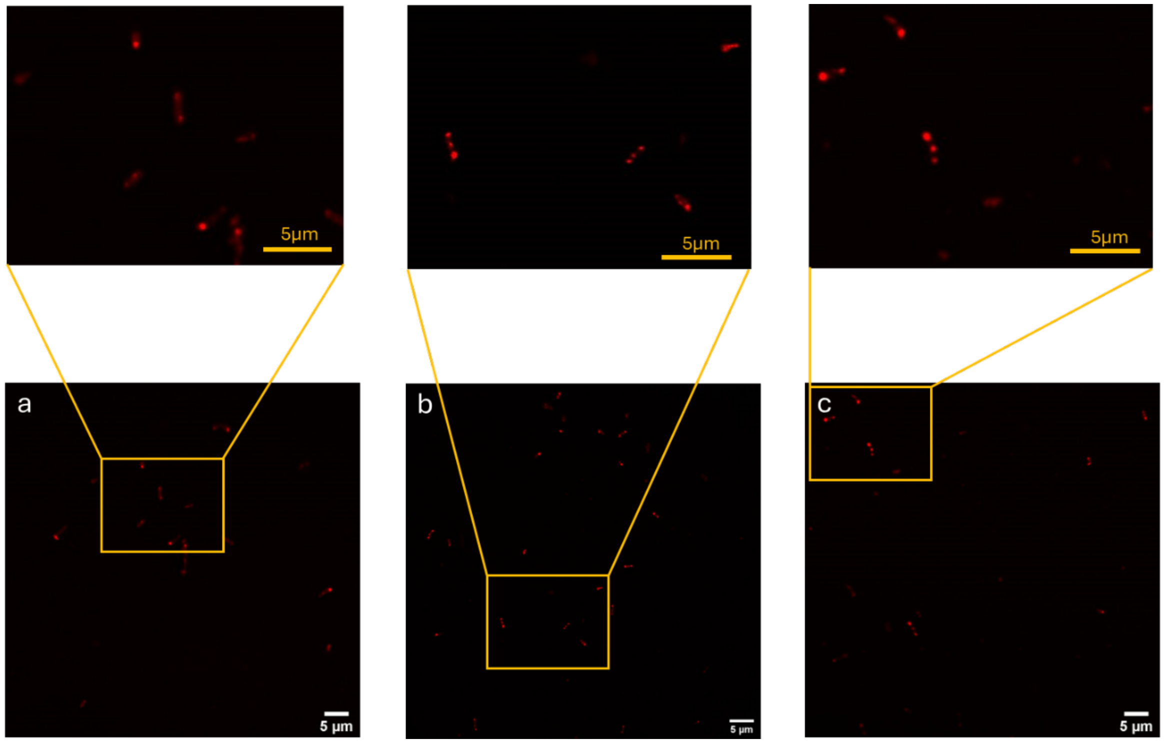

Figure S6. PHA localization in *B. diazoefficiens* USDA 110 grown in liquid culture (a), and in soybean nodules (b), and USDA110 *spc4* grown in nodules (c). Broth-grown cells have terminal PHA granules, while nodule-grown bacteroids contain PHA dispersed across the cell.

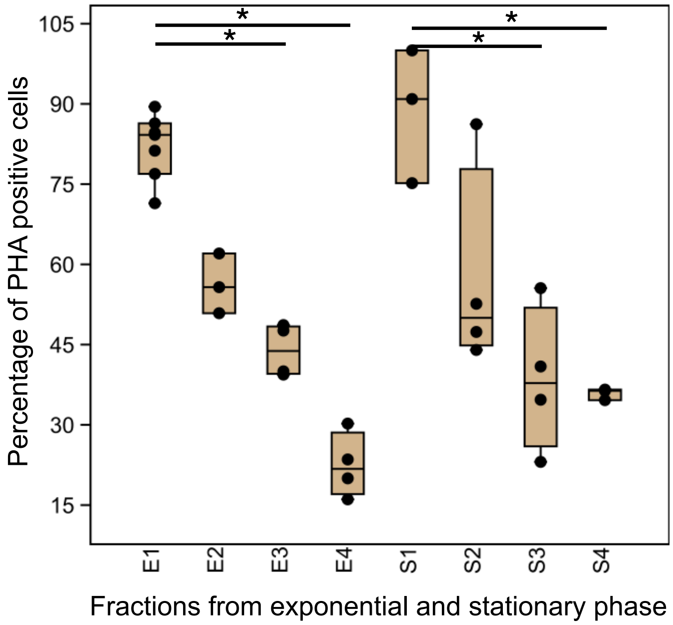

Figure S7. Percentage of PHA positive cells in exponential and stationary phase fractions. Cells with at least one red fluorescent domain were counted as PHA positive, and were counted on at least 3 fields, with a minimum of 100 cells (Dunn's post hoc with $p < 0.05$).

References

Sandhu AK, Brown MR, Subramanian S, Brözel VS (2023) *Bradyrhizobium diazoefficiens* USDA 110 displays plasticity in the attachment phenotype when grown in different soybean root exudate compounds. Frontiers in Microbiology 14: 1190396.

Vilain S, Pretorius JM, Theron J, Brözel VS (2009) DNA as an adhesin: *Bacillus cereus* requires extracellular DNA to form biofilms. Applied and environmental microbiology 75: 2861-2868.
